# Supplementary material for: Modeling COVID-19 disease processes by remote elicitation of causal Bayesian networks from medical experts
Source: BMC Med Res Methodol. 2023 Mar 29;23:76. doi: 10.1186/s12874-023-01856-1 (PMC10050813; doi:10.1186/s12874-023-01856-1)

***Respiratory* causal DAG v3.8**

Additional file prepared for Mascaro et al. (2022); reuse freely with acknowledgement.

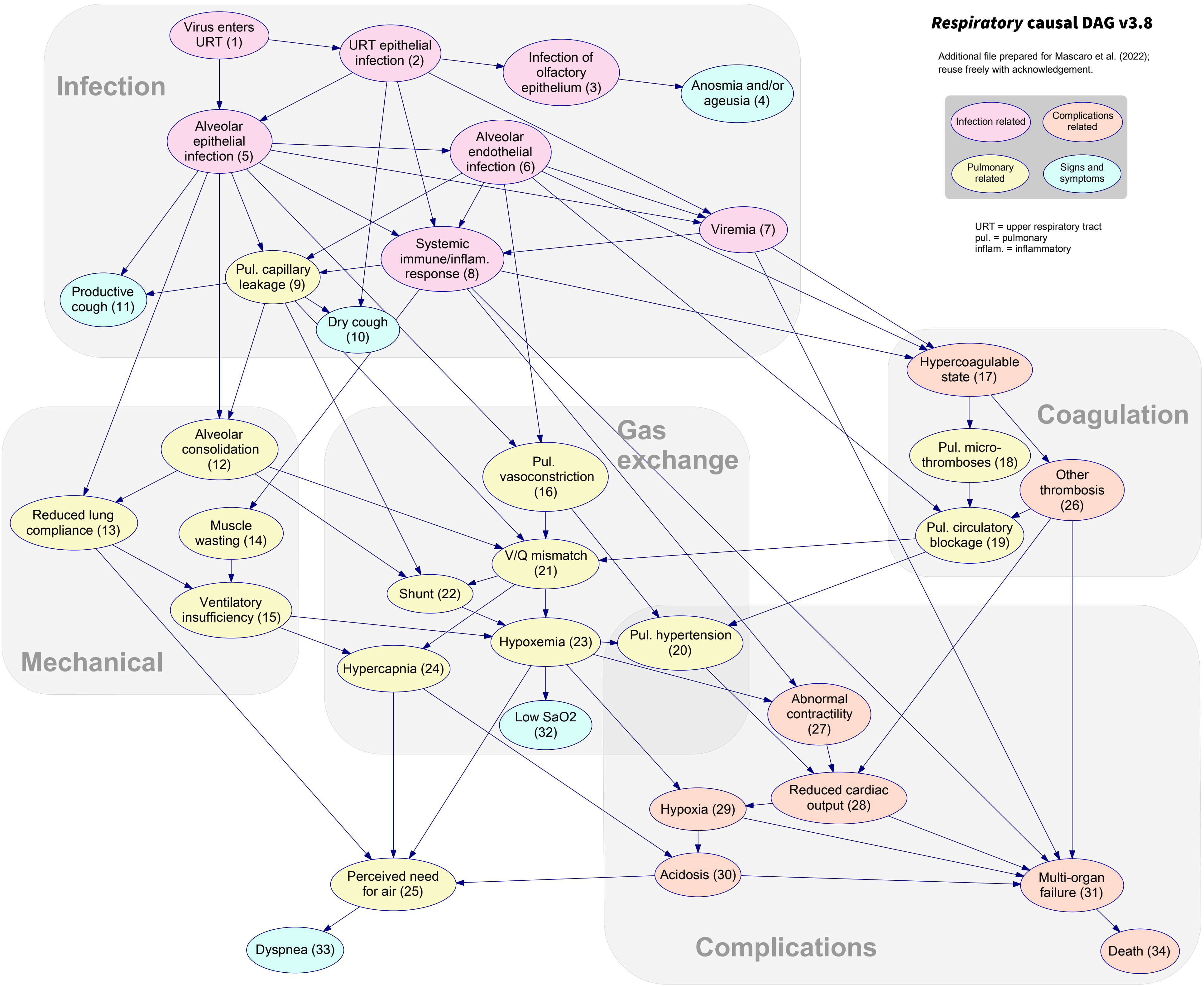

Supplement: Supplementary file 1 — All the following additional files will remain available on our OSF project page [14] in the versions referred to here, accompanied by any subsequent versions of them that we develop. Additional file 1.Respiratory causal DAG v3.8. This depicts the initial pathophysiological process of SARS-CoV-2 in the respiratory system, outlining multiple and often concurrent pathways from viral infection to key downstream complications such as multi-organ failure. Some variables are latent (i.e., not directly observable) but their probability distributions can be inferred from observable evidence such as clinical signs, symptoms and laboratory measurements, not all of which are shown in the diagram. Many mechanisms described in the BN can be influenced by background factors such as age, sex, and comorbidities, which are also not shown. BNs are acyclic, so feedback loops that may occur as the disease progresses are not included in the diagram. We divide the nodes into four color-coded categories: Infection process (pink), Pulmonary details (yellow), resulting Complications (orange), and a few illustrative examples of Signs and symptoms (cyan). Within the pulmonary system, we distinguish (using background boxes) three pathways from Infection to possible Complications: involving problems with Mechanical operation of the lungs, Gas exchange, and Coagulation. [file 12874_2023_1856_MOESM1_ESM.pdf]
