# Supplementary material for: Modeling COVID-19 disease processes by remote elicitation of causal Bayesian networks from medical experts
Source: BMC Med Res Methodol. 2023 Mar 29;23:76. doi: 10.1186/s12874-023-01856-1 (PMC10050813; doi:10.1186/s12874-023-01856-1)
Supplement: Supplementary file 2 — Additional file 2.Complications causal DAG v3.8. This depicts the main physiological processes underlying the progression of COVID-19 from the initial infection in the Pulmonary system (nodes in yellow) to complications in other organs. Vascular (nodes in blue) and Cardiac (nodes in green) systems are modeled in more detail due to their likely earlier involvement and greater system-wide impact on Other Organs (nodes in orange), i.e., liver, kidney, hematologic, gastrointestinal, cortical and brainstem dysfunction. Mechanisms that have a system-wide impact are colored in off-white, and we include two illustrative examples of background factors (nodes in purple). [file 12874_2023_1856_MOESM2_ESM.pdf]

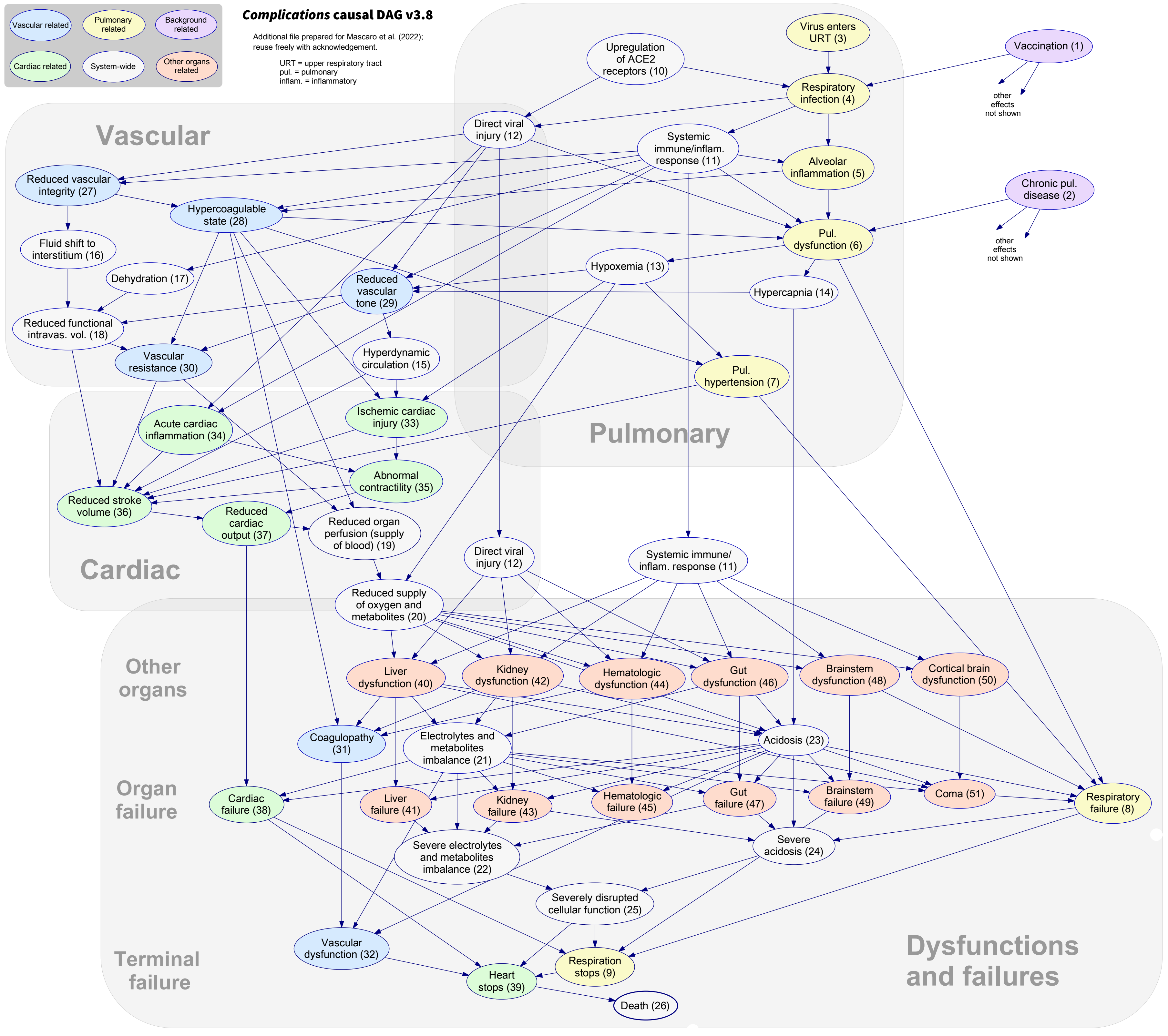

Vascular related
Pulmonary related
Background related
Cardiac related
System-wide
Other organs related
Complications causal DAG v3.8
Additional file prepared for Mascaro et al. (2022); reuse freely with acknowledgement.
URT = upper respiratory tract
pul. = pulmonary
inflam. = inflammatory
Vascular
Pulmonary
Cardiac
Other organs
Organ failure
Terminal failure
Dysfunctions and failures
Virus enters URT (3)
Vaccination (1)
other effects not shown
Upregulation of ACE2 receptors (10)
Respiratory infection (4)
Direct viral injury (12)
Systemic immune/inflam. response (11)
Alveolar inflammation (5)
Chronic pul. disease (2)
other effects not shown
Reduced vascular integrity (27)
Hypercoagulable state (28)
Pul. dysfunction (6)
Fluid shift to interstitium (16)
Dehydration (17)
Hypoxemia (13)
Reduced vascular tone (29)
Hypercapnia (14)
Reduced functional intravas. vol. (18)
Vascular resistance (30)
Hyperdynamic circulation (15)
Pul. hypertension (7)
Acute cardiac inflammation (34)
Ischemic cardiac injury (33)
Abnormal contractility (35)
Reduced stroke volume (36)
Reduced cardiac output (37)
Reduced organ perfusion (supply of blood) (19)
Direct viral injury (12)
Systemic immune/ inflam. response (11)
Reduced supply of oxygen and metabolites (20)
Liver dysfunction (40)
Kidney dysfunction (42)
Hematologic dysfunction (44)
Gut dysfunction (46)
Brainstem dysfunction (48)
Cortical brain dysfunction (50)
Coagulopathy (31)
Electrolytes and metabolites imbalance (21)
Acidosis (23)
Cardiac failure (38)
Liver failure (41)
Kidney failure (43)
Hematologic failure (45)
Gut failure (47)
Brainstem failure (49)
Coma (51)
Respiratory failure (8)
Severe electrolytes and metabolites imbalance (22)
Severe acidosis (24)
Severely disrupted cellular function (25)
Vascular dysfunction (32)
Respiration stops (9)
Heart stops (39)
Death (26)
