# Supplementary material for: Modeling COVID-19 disease processes by remote elicitation of causal Bayesian networks from medical experts
Source: BMC Med Res Methodol. 2023 Mar 29;23:76. doi: 10.1186/s12874-023-01856-1 (PMC10050813; doi:10.1186/s12874-023-01856-1)
Supplement: Supplementary file 3 — Additional file 3.Respiratory BN dictionary v3.8. This table for the Respiratory BN specifies, for each variable, a description of the variable and its relationships to its parent nodes, supported by references to academic literature listed in a bibliography. Relevant evidence, background factors, and some feedback loops are noted even if not included in the BN diagram. [file 12874_2023_1856_MOESM3_ESM.pdf]

Respiratory BN dictionary v3.8

Additional file prepared for Mascaro et al (2022); reuse freely with acknowledgement.

ID	Variable name	Description	Parent nodes	Relationships with parent nodes
1	**Virus enters upper respiratory tract (URT)**	SARS-CoV-2 viral particles inhaled and attach to upper respiratory tract mucosal surface. The size of the viral inoculum is dependent on exposure related factors, not included in the current model.	None	NA
2	**Upper respiratory tract (URT) epithelial infection**	Viral infection of upper respiratory tract epithelial cells +/- signaling an immune response and leading to local inflammation	Virus enters upper respiratory tract	SARS-CoV-2 infection begins in the upper respiratory tract [1] causing infection of the epithelial cells in the upper respiratory tract.
3	**Infection of olfactory epithelium**	Viral infection of the olfactory epithelial cells +/- leading to impaired olfaction	Upper respiratory tract epithelial infection	SARS-CoV-2 infects the olfactory epithelium by contiguous spread, entering via the ACE2 receptors [2], [3].
4	**Ageusia and/or anosmia**	Loss of the ability to taste and/or smell	Infection of supporting olfactory epithelium	The sensation of loss of smell and/or taste occurs due damage caused by infection of the cells supporting the olfactory neurons, the olfactory epithelial cells [2].
5	**Alveolar epithelial infection**	Viral infection of the alveolar cells, +/- inducing an immune response which leads to local inflammation.	Virus enters upper respiratory tract, Upper respiratory tract epithelial infection	The upper airways acts as a portal for SARS-CoV-2 entry and infection of the lower airways [4]. This may occur by contiguous spread, or by aspiration of virus from the upper airway into the lower airway.
6	**Alveolar endothelial infection**	Viral infection of the endothelial cells of the capillaries of the terminal airways, +/- inducing an immune response which leads to local inflammation.	Alveolar epithelial infection	SARS-CoV-2 directly transverses the alveolar epithelium to infect the alveolar endothelium [5].
7	**Viremia**	Presence of SARS-CoV-2 in blood allowing for systemic dissemination of the virus.	Upper respiratory tract epithelial infection, Alveolar epithelial infection, Alveolar endothelial infection	In COVID-19, the barrier between the respiratory tract (upper respiratory tract epithelium, alveolar epithelium) and circulatory system (alveolar endothelium) may be compromised causing entry of the virus into the bloodstream [6].
8	**Systemic immune/ inflammatory (inflam.) response**	Activation of innate and/or adaptive immune system by the presence of virus at one or more body site/s. Manifest by the release of pro- +/- anti-inflammatory markers in blood by immune-related cells.	Upper respiratory tract epithelial infection, Alveolar epithelial infection, Alveolar endothelial infection, Viremia	Infection of the alveolar epithelial cells [7], alveolar endothelial cells [8], [9], and/or the presence of viral material in the bloodstream [10] stimulate a systemic inflammatory immune response including the migration of immune cells to the infected site and the release of pro-inflmmatory cytokines and chemokines.

ID	Variable name	Description	Parent nodes	Relationships with parent nodes
9	**Pulmonary capillary leakage**	Leakage of plasma from the pulmonary capillaries into the terminal airways.	Alveolar epithelial infection, Alveolar endothelial infection, Systemic immune/ inflam. response	Infection of the alveoli and/or the alveolar endothelial cells [5] and its associated immune response [11], [12] damage cells resulting in increased vascular permeability and leakage of plasma into the tissues.
10	**Dry cough**	Cough without the production of mucus or phlegm.	Pulmonary capillary leakage, Upper respiratory tract epithelial infection	Upper respiratory tract inflammation can trigger cough in the absence of mucus production. Inflammation and irritation of the lower respiratory tract can also trigger cough. [13]
11	**Productive cough**	Cough with the production of mucus or phlegm.	Alveolar epithelial infection, Pulmonary capillary leakage	Infection and inflammation of the lower airways causes mucus production. The presence of secretions in the lower respiratory tract triggers cough as a reflex to remove the secretions in order to maintain ventilation. [13], [14]
12	**Alveolar consolidation**	Filling of alveolar airspaces with fluid, cells, or other susbstances.	Alveolar epithelial infection, Pulmonary capillary leakage.	In response to infection of the alveolar epithelial cells and increased permeability of the capillaries, plasma leaks from the capillaries and fills the terminal airspaces. Cell death further stimulates the infiltration of the terminal airspaces with immune cells and a protein-rich exudate [15], [16].
13	**Reduced lung compliance**	Reduced ability of the lungs to expand (increase in volume) per unit increase in inspiratory (transpulmonary) pressure.	Alveolar consolidation, Alveolar epithelial infection	Infection of type II pneumocytes of the alveolar epithelium reduces surfactant secretion causing atelectasis of the lungs which, along with alveolar consolidation, increases the inspiratory pressure required for inspiration [15]
14	**Muscle wasting**	Loss of respiratory skeletal muscle mass resulting in a functional respiratory impairment.	Systemic immune/inflam. response	Systemic inflammation and hypoxia (feedback loop not shown) drives catabolism of skeletal muscle and decreases muscle protein synthesis that eventually causes muscle wasting and reduced mechanical capacity for the work of breathing [17]. The loss of skeletal muscle mass can also be caused by immobility, aging, malnutrition, medications (not shown in this model). Note that this a slower process that occur over weeks rather than days (compared with some other events described in the model).
15	**Ventilatory insufficiency**	The collective chemical and mechanical factors preventing the adequate oxygenation of blood and elimination of carbon dioxide.	Reduced lung compliance, Muscle wasting	When alveolar consolidation reaches a certain magnitude, there is a decrease in gas volume in the lungs (due to reduced maximum volume and reduced compliance), and tidal volumes produced for a given inspiratory pressure decline preventing the exchange of oxygen and carbon dioxide [18].

ID	Variable name	Description	Parent nodes	Relationships with parent nodes
16	**Pulmonary vasoconstriction**	Localised or widespread constriction of the pulmonary vasculature in response to low alveolar oxygen or molecular promotors resulting in increased vascular resistance.	Alveolar epithelial infection, Alveolar endothelial infection	Alveolar epithelial and endothelial infection lead to local hypoxia (feedback loop not shown) that can cause vasoconstriction. Disruption in the renin-angiotensin system associated with endothelial infection via ACE2 receptors reduces expression of ACE2, which results in an increase of a number of molecules that promote pulmonary vasoconstriction [19].
17	**Hypercoagulable state**	Increased propensity of the blood to coagulate.	Systemic immune/inflam. response, Alveolar endothelial infection, Viremia	Presence of viral material in the blood [10], infection of endothelial cells [20] and the systemic immune/inflammatory response [21] activate the coagulation cascade (thrombin/ coagulation proteases, fibrinogen and platelets) inducing a hypercoagulable state as a physiological effort to repair damaged blood vessels and limit viral replication [22].
18	**Pulmonary microthrombosis**	Small fragments of coagulated blood in the pulmonary vasculature.	Hypercoagulable state	Coagulation is initiated as a result of the hypercoagulable state and pulmonary endothelial injury resulting in disruption of the endothelial cell membrane manifesting a pulmonary microthrombi [23].
19	**Pulmonary circulatory blockage**	Obstruction of pulmonary circulation to blood flow.	Alveolar endothelial infection, Pulmonary microthrombosis, Other thrombosis	The formation of multiple microthrombi and/or larger thrombi cause emboli in the pulmonary arteries [24]. Alveolar inflammation and blood clots can lead to blockage of pulmonary circulation.
20	**Pulmonary hypertension**	Elevation of the blood pressure of the pulmonary vasculature.	Pulmonary circulatory blockage, Pulmonary vasoconstriction, Hypoxemia	Vascular manifestations such as pulmonary and other thrombosis and sustained pulmonary vasoconstriction (which can be caused by hypoxemia) result in pulmonary hypertension due to persistently high blood pressure [21], [25].
21	**V/Q mismatch**	A state whereby the ability of lungs to exchange gas in some parts of the lungs is poorly matched to the perfusion of those parts of the lung with blood.	Pulmonary circulatory blockage, Pulmonary vasoconstriction, Pulmonary capillary leakage, Alveolar consolidation	Ventilation/ perfusion (V/Q) mismatch can result from obstruction of circulation in the pulmonary arteries due to pulmonary thrombosis/ embolism, impeding blood flow and gas exchange [26], [27]. Consolidation of the terminal airspaces due to inflammation and alveolar capillary leak reduces surface area available for gas exchange [28]. Vasoconstriction helps to match regional perfusion to ventilation in the lungs. The failure of the hypoxic pulmonary vasoconstriction mechanism leads to persistent high pulmonary blood flow of poorly ventilated alveoli [29] which allows for carbon dioxide to be removed at a greater rate than the receipt of oxygen.
22	**Shunt**	An extreme state of V/Q mismatch where part of the pulmonary circulation bypasses ventilated lung.	Pulmonary capillary leakage, Alveolar consolidation, V/Q mismatch	Complete absence of ventilation due to a pulmonary capillary leakage and consolidation, and failure of local compensatory pulmonary vasoconstriction, allows for perfusion of blood to areas with no ventilation, resulting in extreme V/Q mismatch [30]
23	**Hypoxemia**	Reduced concentration of oxygen in the blood.	V/Q mismatch, Shunt, Ventilatory insufficiency	Oxygen levels in the blood are reduced due to inadequate gas exchange, V/Q mismatch or intrapulmonary shunting due to persistent pulmonary arterial blood flow to non-ventilated alveoli [28], [29]

ID	Variable name	Description	Parent nodes	Relationships with parent nodes
24	**Hypercapnia**	Increased concentration of carbon dioxide in the blood.	V/Q mismatch, Ventilatory insufficiency	Carbon dioxide may accumulate in the blood due to insufficient ventilation and inadequate gas exchange [28], [29].
25	**Perceived need for air**	Sensation of shortness of breath.	Reduced lung compliance, Hypoxemia, Hypercapnia, Acidosis	Sensation of insufficient respiration (oxygen) due to detection of low oxygen and/or high carbon dioxide concentrations and/or low pH by peripheral and central chemoreceptors. Mechanostretch receptors in the muscles and lungs also provide sensory feedback as a result of reduced compliance and respiratory muscle weakness due to altered lung mechanics [29].
26	**Other thrombosis**	Large thromboses including pulmonary arterial thrombosis and thrombosis of the large veins.	Hypercoagulable state	Large thromboses arise as a complication of the hypercoagulable state [31].
27	**Abnormal contractility**	In this model, refers to abnormal cardiac electrophysiology, resulting in an alteration in the rate and/or coordination of contractility.	Systemic immune/inflam. response, Hypoxemia	Hypoxemia and pro-inflammatory cytokines can impair the function of myocardial cells [21], [32].
28	**Reduced cardiac output**	A reduction in the volume of blood the heart pumps per unit time.	Abnormal contractility, Other thrombosis, Pulmonary hypertension	Cardiac output is a function of both the heart rate and stroke volume. Coronary thrombosis can reduce stroke volume by causing acute myocardial injury from ischemia, reducing contractility. Myocardial injury may also reduce the heart rate, including by disruption to electrical conduction (heart block) [21], [28] Pulmonary hypertension can impair cardiac output because increased vascular resistance increases the myocardial contractility required for forward flow [33].
29	**Hypoxia**	Reduced concentration of oxygen in blood, either bound and/or unbound to hemoglobin	Hypoxemia, Reduced cardiac output	Low concentration of oxygen in the blood and reduced blood flow prevents adequate oxygen delivery to the tissues [28]
30	**Acidosis**	Increased acidity of the blood and body tissues, altering blood oxygen carrying capacity and muscle function, including cardiac contractility.	Hypercapnia, Hypoxia	Respiratory acidosis develops due to retention of carbon dioxide resulting in a decrease in pH [34]. Hypoxia causes cells to utilise anaerobic metabolism resulting in a decrease in pH.
31	**Multiorgan failure**	Altered function of two or more organ systems and failure to maintain homeostasis.	Hypoxia, Acidosis, Other thrombosis, Viremia, Systemic immune/inflam. response, Reduced cardiac output	Viral infection and systemic inflammatory responses can impair organ function via cellular damage. Sustained hypoxia and acidosis cause cell death and subsequent organ failure [28][35]. Hypoxia and acidosis can be caused by reduced blood flow to organs due to impaired cardiac output and reduced perfusion due to thrombosis.

ID	Variable name	Description	Parent nodes	Relationships with parent nodes
32	**Low oxygen saturation (SaO2)**	Low proportion of hemoglobin bound to oxygen.	Hypoxemia	Reduced oxygen concentrations in blood causes a reduction in the proportion of hemoglobin with is bound to oxygen which can be measured by pulse oximetry [28].
33	**Dyspnea**	A subjective (reported) experience of breathing discomfort (shortness of breath).	Perceived need for air	Dyspnea occurs when an individual responds to the sensation of need for oxygen (e.g., hypoxemia) [29].
34	**Death**	Mortality due to COVID-19	Multi-organ failure	Sustained multi-organ failure causes respiratory and cardiac arrest [36].

Selected References

[1] L. Zou *et al.*, "SARS-CoV-2 Viral Load in Upper Respiratory Specimens of Infected Patients," *N. Engl. J. Med.*, Feb. 2020, doi: 10.1056/NEJMc2001737.

[2] J. Meinhardt *et al.*, "Olfactory transmucosal SARS-CoV-2 invasion as a port of central nervous system entry in individuals with COVID-19," *Nat. Neurosci.*, vol. 24, no. 2, Art. no. 2, Feb. 2021, doi: 10.1038/s41593-020-00758-5.

[3] D. H. Brann *et al.*, "Non-neuronal expression of SARS-CoV-2 entry genes in the olfactory system suggests mechanisms underlying COVID-19-associated anosmia," *Sci. Adv.*, vol. 6, no. 31, Jul. 2020, doi: 10.1126/sciadv.abc5801.

[4] K. P. Y. Hui *et al.*, "Tropism, replication competence, and innate immune responses of the coronavirus SARS-CoV-2 in human respiratory tract and conjunctiva: an analysis in ex-vivo and in-vitro cultures," *Lancet Respir. Med.*, vol. 8, no. 7, pp. 687–695, Jul. 2020, doi: 10.1016/S2213-2600(20)30193-4.

[5] L.-A. Teuwen, V. Geldhof, A. Pasut, and P. Carmeliet, "COVID-19: the vasculature unleashed," *Nat. Rev. Immunol.*, pp. 1–3, May 2020, doi: 10.1038/s41577-020-0343-0.

[6] P. Wang *et al.*, "A cross-talk between epithelium and endothelium mediates human alveolar–capillary injury during SARS-CoV-2 infection," *Cell Death Dis.*, vol. 11, no. 12, Art. no. 12, Dec. 2020, doi: 10.1038/s41419-020-03252-9.

[7] H. Chu *et al.*, "Comparative Replication and Immune Activation Profiles of SARS-CoV-2 and SARS-CoV in Human Lungs: An Ex Vivo Study With Implications for the Pathogenesis of COVID-19," *Clin. Infect. Dis.*, vol. 71, no. 6, pp. 1400–1409, Sep. 2020, doi: 10.1093/cid/ciaa410.

[8] L. C. Barbosa, T. L. Gonçalves, L. P. de Araujo, L. V. de O. Rosario, and V. P. Ferrer, "Endothelial cells and SARS-CoV-2: An intimate relationship," *Vascul. Pharmacol.*, Jan. 2021, doi: 10.1016/j.vph.2021.106829.

[9] Z. Varga *et al.*, "Endothelial cell infection and endotheliitis in COVID-19," *The Lancet*, vol. 395, no. 10234, pp. 1417–1418, May 2020, doi: 10.1016/S0140-6736(20)30937-5.

[10] J. F. Bermejo-Martin *et al.*, "Viral RNA load in plasma is associated with critical illness and a dysregulated host response in COVID-19," *Crit. Care*, vol. 24, no. 1, p. 691, Dec. 2020, doi: 10.1186/s13054-020-03398-0.

[11] M. Bahloul *et al.*, "Pulmonary capillary leak syndrome following COVID-19 virus infection," *J. Med. Virol.*, vol. n/a, no. n/a, doi: 10.1002/jmv.26152.

[12] S. Pons, S. Fodil, E. Azoulay, and L. Zafrani, "The vascular endothelium: the cornerstone of organ dysfunction in severe SARS-CoV-2 infection," *Crit. Care*, vol. 24, Jun. 2020, doi: 10.1186/s13054-020-03062-7.

[13] E. Begic, Z. Begic, A. Dobraca, and E. Hasanbegovic, "Productive Cough in Children and Adolescents – View from Primary Health Care System," *Med. Arch.*, vol. 71, no. 1, pp. 66–68, Feb. 2017, doi: 10.5455/medarh.2017.71.66-68.

[14] M. J. Martin and T. W. Harrison, "Causes of chronic productive cough: An approach to management," *Respir. Med.*, vol. 109, no. 9, pp. 1105–1113, Sep. 2015, doi: 10.1016/j.rmed.2015.05.020.

[15] T. L. Oliveira *et al.*, "Pathophysiology of SARS-CoV-2 in Lung of Diabetic Patients," *Front. Physiol.*, vol. 11, 2020, doi: 10.3389/fphys.2020.587013.

[16] T. Schaller *et al.*, "Postmortem Examination of Patients With COVID-19," *JAMA*, May 2020, doi: 10.1001/jama.2020.8907.

[17] M. C. de Andrade-Junior, I. C. D. de Salles, C. M. M. de Brito, L. Pastore-Junior, R. F. Righetti, and W. P. Yamaguti, "Skeletal Muscle Wasting and Function Impairment in Intensive Care Patients With Severe COVID-19," *Front. Physiol.*, vol. 12, 2021, doi: 10.3389/fphys.2021.640973.

[18] P. Pelosi, L. D'Andrea, G. Vitale, A. Pesenti, and L. Gattinoni, "Vertical gradient of regional lung inflation in adult respiratory distress syndrome," *Am. J. Respir. Crit. Care Med.*, vol. 149, no. 1, pp. 8–13, Jan. 1994, doi: 10.1164/ajrccm.149.1.8111603.

[19] H. Karmouty-Quintana, R. A. Thandavarayan, S. P. Keller, S. Sahay, L. M. Pandit, and B. Akkanti, "Emerging Mechanisms of Pulmonary Vasoconstriction in SARS-CoV-2-Induced Acute Respiratory Distress Syndrome (ARDS) and Potential Therapeutic Targets," *Int. J. Mol. Sci.*, vol. 21, no. 21, Oct. 2020, doi: 10.3390/ijms21218081.

[20] M. Dolhnikoff *et al.*, "Pathological evidence of pulmonary thrombotic phenomena in severe COVID-19," *J. Thromb. Haemost.*, vol. n/a, no. n/a, doi: 10.1111/jth.14844.

[21] F. Potus *et al.*, "Novel insights on the pulmonary vascular consequences of COVID-19," *Am. J. Physiol. - Lung Cell. Mol. Physiol.*, vol. 319, no. 2, pp. L277–L288, Aug. 2020, doi: 10.1152/ajplung.00195.2020.

[22] Y. Sakr *et al.*, "The clinical spectrum of pulmonary thromboembolism in patients with coronavirus disease-2019 (COVID-19) pneumonia: A European case series," *J. Crit. Care*, vol. 61, pp. 39–44, Feb. 2021, doi: 10.1016/j.jcrc.2020.09.021.

[23] M. Ackermann *et al.*, "Pulmonary Vascular Endothelialitis, Thrombosis, and Angiogenesis in Covid-19," *N. Engl. J. Med.*, vol. 0, no. 0, p. null, May 2020, doi: 10.1056/NEJMoa2015432.

[24] Y. J. Suh *et al.*, "Pulmonary Embolism and Deep Vein Thrombosis in COVID-19: A Systematic Review and Meta-Analysis," *Radiology*, vol. 298, no. 2, pp. E70–E80, Dec. 2020, doi: 10.1148/radiol.2020203557.

[25] R. T. Dhawan *et al.*, "Beyond the clot: perfusion imaging of the pulmonary vasculature after COVID-19," *Lancet Respir. Med.*, vol. 9, no. 1, pp. 107–116, Jan. 2021, doi: 10.1016/S2213-2600(20)30407-0.

[26] F. Scudiero *et al.*, "Pulmonary embolism in COVID-19 patients: prevalence, predictors and clinical outcome," *Thromb. Res.*, vol. 198, pp. 34–39, Feb. 2021, doi: 10.1016/j.thromres.2020.11.017.

[27] G. Grasselli *et al.*, "Pathophysiology of COVID-19-associated acute respiratory distress syndrome: a multicentre prospective observational study," *Lancet Respir. Med.*, vol. 8, no. 12, pp. 1201–1208, Dec. 2020, doi: 10.1016/S2213-2600(20)30370-2.

[28] P. E. Bickler, J. R. Feiner, M. S. Lipnick, and W. McKleroy, "'Silent' Presentation of Hypoxemia and Cardiorespiratory Compensation in COVID-19," *Anesthesiology*, Sep. 2020, doi: 10.1097/ALN.0000000000003578.

[29] S. Dhont, E. Derom, E. Van Braeckel, P. Depuydt, and B. N. Lambrecht, "The pathophysiology of 'happy' hypoxemia in COVID-19," *Respir. Res.*, vol. 21, no. 1, p. 198, Jul. 2020, doi: 10.1186/s12931-020-01462-5.

[30] M. Lang *et al.*, "Hypoxaemia related to COVID-19: vascular and perfusion abnormalities on dual-energy CT," *Lancet Infect. Dis.*, vol. 20, no. 12, pp. 1365–1366, Dec. 2020, doi: 10.1016/S1473-3099(20)30367-4.

[31] A. Kichloo *et al.*, "COVID-19 and Hypercoagulability: A Review," *Clin. Appl. Thromb.*, vol. 26, p. 1076029620962853, Jan. 2020, doi: 10.1177/1076029620962853.

[32] S. Zaim, J. H. Chong, V. Sankaranarayanan, and A. Harky, "COVID-19 and Multiorgan Response," *Curr. Probl. Cardiol.*, vol. 45, no. 8, p. 100618, Aug. 2020, doi: 10.1016/j.cpcardiol.2020.100618.

[33] Y.-C. Lai, K. C. Potoka, H. C. Champion, A. L. Mora, and M. T. Gladwin, "Pulmonary Arterial Hypertension: The Clinical Syndrome," *Circ. Res.*, vol. 115, no. 1, pp. 115–130, Jun. 2014, doi: 10.1161/CIRCRESAHA.115.301146.

[34] B. Akkanti *et al.*, "Physiologic Improvement in Respiratory Acidosis Using Extracorporeal Co2 Removal With Hemolung Respiratory Assist System in the Management of Severe Respiratory Failure From Coronavirus Disease 2019," *Crit. Care Explor.*, vol. 3, no. 3, p. e0372, Mar. 2021, doi: 10.1097/CCE.0000000000000372.

[35] Z. O. Serebrovska, E. Y. Chong, T. V. Serebrovska, L. V. Tumanovska, and L. Xi, "Hypoxia, HIF-1α, and COVID-19: from pathogenic factors to potential therapeutic targets," *Acta Pharmacol. Sin.*, vol. 41, no. 12, Art. no. 12, Dec. 2020, doi: 10.1038/s41401-020-00554-8.

[36] Y.-J. Sun, Y.-J. Feng, J. Chen, B. Li, Z.-C. Luo, and P.-X. Wang, "Clinical Features of Fatalities in Patients With COVID-19," *Disaster Med. Public Health Prep.*, pp. 1–3, undefined/ed, doi: 10.1017/dmp.2020.235.
