# Supplementary material for: Modeling COVID-19 disease processes by remote elicitation of causal Bayesian networks from medical experts
Source: BMC Med Res Methodol. 2023 Mar 29;23:76. doi: 10.1186/s12874-023-01856-1 (PMC10050813; doi:10.1186/s12874-023-01856-1)
Supplement: Supplementary file 4 — Additional file 4.Complications BN dictionary v3.8. This table for the Complications BN specifies, for each variable, a description of the variable and its relationships to its parent nodes, supported by references to academic literature listed in a bibliography. Relevant evidence, background factors, and some feedback loops are noted even if not included in the BN diagram. [file 12874_2023_1856_MOESM4_ESM.pdf]

Complications BN dictionary v3.8

Additional file prepared for Mascaro et al (2022); reuse freely with acknowledgement.

ID	Variable name	Description	Parent nodes	Relationships with parent nodes
1	**Vaccination**	Vaccinated against SARS-CoV-2.	None	NA
2	**Chronic pulmonary disease**	Chronic pulmonary disease is recorded in medical history.	None	NA
3	**Virus enters upper respiratory tract (URT)**	SARS-CoV-2 viral particles inhaled and attach to upper respiratory tract mucosal surface. The size of the viral inoculum is dependent on exposure related factors, not included in the current model.	None	NA
4	**Respiratory infection**	Viral replication occurring at one or more sites of the respiratory tract, primarily initiating in the nasopharynx.	Virus enters upper respiratory tract, Upregulation of ACE2 receptors	SARS-CoV-2 infects human cells by binding with angiotensin converting enzyme-2 (ACE-2), which is highly expressed on nasal epithelial cells [1].
5	**Alveolar inflammation**	Virus infects the terminal airways inducing an immune response which causes local inflammation.	Respiratory infection, Systemic immune/ inflam. response	The upper airway acts as a portal for SARS-CoV-2 entry and infection of the terminal airway epithelium and endothelium [2], [3].
6	**Pulmonary dysfunction**	Impaired capacity of the lungs to oxygenate blood and/or eliminate carbon dioxide (gas exchange).	Direct viral injury, Systemic immune/ inflam. response, Alveolar inflammation, Hypercoagulable state	Gas exchange can be impaired by one or more of injury of alveolar cells, consolidation of the terminal airspaces due to pulmonary capillary leakage (caused by direct viral injury and/or immune-mediated damage), reduced pulmonary perfusion caused by circulatory blockage (due to microthrombosis), and ventilatory insufficiency (due to reduced pulmonary compliance and/or respiratory effort). (See details in the respiratory model)
7	**Pulmonary hypertension**	Elevated blood pressure of the pulmonary vasculature.	Hypercoagulable state, Hypoxemia	Pulmonary hypertension can result from obstruction of the pulmonary vasculature due to thrombosis (pulmonary microthrombosis and/or emboli), and/or pulmonary vasoconstriction (triggered by local hypoxemia) [4]–[6].
8	**Respiratory failure**	Inability of the lungs to meet the body's basic requirements for gas-exchange.	Pulmonary dysfunction, Pulmonary hypertension, brainstem dysfunction, Acidosis, Coma	Pulmonary dysfunction and pulmonary hypertension impair gas exchange. Hypoventilation caused by brain dysfunction and coma can also reduce the concentration of oxygen and increases the concentration of carbon dioxide in the terminal airways, leading to reduced gas exchange. Acidosis increases the body's need for gas exchange by increasing amount of carbon dioxide that needs to be exchanged in order to resolve the acidosis. [7]

ID	Variable name	Description	Parent nodes	Relationships with parent nodes
9	**Respiration stops**	Respiratory arrest.	Cardiac failure, Brainstem failure, Severely disrupted cellular function, Respiratory failure	Progressive hypoxemia and hypercapnia severely disrupt cellular functions, brainstem function and eventual loss of respiratory drive.
10	**Upregulation of ACE2 receptors**	Expression of angiotensin-converting enzyme 2 (ACE2) protein on the surface of cells, including in the nasal epithelium, lungs, arteries, heart, kidney, and intestines.	None	NA
11	**Systemic immune/ inflammatory (inflam.) response**	Activation of innate and/or adaptive immune system by the presence of virus at one or more body site/s. Manifest by the release of pro- +/- anti-inflammatory markers in blood by immune-related cells.	Respiratory infection	Viral infection stimulates systemic immune and inflammatory response [2], [3], [8].
12	**Direct viral injury**	Injury to cells caused by the direct effect of viral infection (i.e. independent of immune-mediated injury).	Respiratory infection, Upregulation of ACE2 receptors	Viral infection of cells may cause direct damage to cells. Upregulation of ACE2 receptors facilitates viral entry, contributing to further direct cellular damage [9], [10].
13	**Hypoxemia**	Reduced concentration of oxygen in blood, either bound and/or unbound to hemoglobin	Pulmonary dysfunction	Insufficient gas exchange can reduce blood oxygen concentartion [11], [12].
14	**Hypercapnia**	Increased concentration of carbon dioxide in blood.	Pulmonary dysfunction	Insufficient gas exchange can lead to accumulation of carbon dioxide in blood [11], [12].
15	**Hyperdynamic circulation**	Increased cardiac output above baseline to maintain tissue perfusion, for example in response to inappropriately low peripheral vascular resistance.	Reduced vascular tone	Inappropriately low peripheral vascular tone can clinically manifest as a hyperdynamic circulation [13].
16	**Fluid shift to interstitium**	Fluid moves from the intravascular space (blood vessels) into the interstitium or "third" space.	Reduced vascular integrity	Increased permeability of the vascular endothelial junction allows plasma to shift into the interstitial space [14].
17	**Dehydration**	A pathological deficiency in total body water.	Systemic immune/inflam. response	The systemic inflammatory response gives rise to fever, increased catabolism, and anorexia which increase fluid losses and reduce fluid intake [15].

ID	Variable name	Description	Parent nodes	Relationships with parent nodes
18	**Reduced functional intravascular volume**	A reduction in the effective volume of circulating blood, including states of absolute decreased intravascular volume due to losses, as well as states of relatively decreased intravascular volume when the capacity of the vascular basin is increased secondary to reduced vascular tone.	Dehydration, Fluid shift to interstitium, Reduced systemic vascular tone	Dehydration and fluid shift result in a reduction in the volume of blood. Reduced vascular tone increases the volume of the vascular basin, reducing the functional volume. [14]
19	**Reduced organ perfusion (supply of blood)**	Reduction in blood flow to organs such as the kidneys and brain.	Hypercoagulable state, Reduced cardiac output, Vascular resistance	Insufficient organ perfusion can be caused insufficient cardiac output, and obstruction to flow from thrombosis (hypercoagulable state) [14]. Decreases in peripheral vascular resistance may reduce blood pressure resulting in reduced organ perfusion [16].
20	**Reduced supply of oxygen and metabolites**	Reduction in supply of oxygen and metabolites for meeting organ needs.	Reduced organ perfusion (supply of blood), Hypoxemia	Decreased perfusion reduces the volume of blood flow and therefore oxygen and metabolites delivered to the organs. Hypoxemia causes insufficient oxygen to meet the organ needs regardless of blood supply.
21	**Electrolyte and metabolites imbalance**	Abnormal blood concentrations of electrolytes, such as sodium, potassium, chloride, calcium, bicarbonate, phosphate, and magnesium	Liver dysfunction, Kidney dysfunction, Gut dysfunction	Abnormal renal function and gastrointestinal losses can adversely alter electrolyte balance through reduced or increased losses respectively. Kidney dysfunction may impair the kidneys electrolyte balancing role. The liver recycles and excretes by-products of metabolism produced by other organs and tissues. These metabolites may be retained when hepatic function is impaired. [17]–[19]
22	**Severe electrolytes and metabolites imbalance**	Pathological abnormality of concentrations of electrolytes.	Electrolytes and metabolites imbalance, Liver failure, Kidney failure, Gut failure	The kidney plays a central role in balancing electrolytes. Kidney failure impairs this homeostatic function. Similar to gut failure, kidney failure may also increase inappropriate losses of electrolytes. [17]–[19]
23	**Acidosis**	Increased acidity of the blood and body tissues, altering blood oxygen carrying capacity and muscle function, including cardiac contractility.	Hypercapnia, Reduced supply of oxygen and metabolites, Kidney dysfunction, Liver dysfunction, Gut dysfunction	The kidneys, gut and respiratory system play critical roles in maintaining acid-base levels in the body. Dysfunction of these systems may result in increased loss of bicarbonate (kidneys and gut) or impaired compensatory mechanisms. Carbon dioxide complexes with bicarbonate in blood to form acid. Low supply of oxygen and metabolites creates a shift to anaerobic metabolism, giving rise to lactic acidosis. The liver helps to clear this lactic acid. [20]
24	**Severe acidosis**	Pathological acidosis of the blood and body tissues exceeding physiological compensatory mechanisms.	Acidosis, Kidney failure, Gut failure, Respiratory failure	The kidneys, gut and respiratory system play critical roles in maintaining acid-base equilibrium in the body. Dysfunction of these systems may result in increased loss of bicarbonate or impaired compensatory mechanisms for respiratory or metabolic acidosis. [21]

ID	Variable name	Description	Parent nodes	Relationships with parent nodes
25	**Severely disrupted cellular function**	Cells unable to perform basic functions	Severe electrolytes and metabolites imbalance, Severe acidosis	Increased acid alters cell metabolism and disturb the cell structure and function. Severe electrolytes and metabolite imbalance impairs cellular metabolic pathways. [22]
26	**Death**	Complete loss of any basic biological functions	Heart stops (asystole).	Cessation of perfusion of organs contributing to cellular dysfunction and death.
27	**Reduced vascular integrity**	Alterations in endothelial structure and function, increasing vascular permeability to plasma +/- blood cells.	Systemic immune/inflam. response, Direct viral injury	Endothelial cells may be damaged either by direct viral injury or indirectly by the systemic immune responses to infection [14].
28	**Hypercoagulable state**	Increased propensity of the blood to coagulate.	Systemic immune/inflam. response, Reduced vascular integrity, Alveolar inflammation	Infection of endothelial cells and the systemic immune/inflammatory response activate the coagulation cascade (thrombin/ coagulation proteases, fibrinogen and platelets) inducing a hypercoagulable state as a physiological effort to repair damaged blood vessels and limit viral replication [4], [23]–[25].
29	**Reduced vascular tone**	Decrease in the ability of the peripheral vasculature to maintain peripheral resistance by vasoconstriction.	Systemic immune response, Direct viral injury, Hypercapnia, Hypoxemia	Endothelial cell death and dysfunction and chemical signals generated as part of the systemic response contribute to the dysregulation of vascular tone [2]. Blood vessels are sensitive to changes in pCO2, the low pH values (hypercapnia) lead to relaxation of vascular smooth muscle [26], [27]. Hypoxemia disrupts the control of vascular tone [28].
30	**Vascular resistance**	Increase or decrease of the resistance of vessel wall.	Reduced vascular tone, Reduced functional intravascular volume, Hypercoagulable state	Tone and vascular resistance can increase in response to reduced functional volume to maintain perfusion. Thrombosis can result in increased vascular resistance and obstruction to downstream blood flow. [14]
31	**Coagulopathy**	Derangement of the balance of the coagulation cascade resulting in increased bleeding with or without blood clotting.	Liver dysfunction, Marrow dysfunction, Kidney dysfunction, Hypercoagulable state	The liver and bone marrow produce components used in clotting and organ dysfunction impairs their production. Uremia (from kidney dysfunction) impairs platelet function and number. Dysfunctional systemic activation of the coagulation pathway can lead to fibrin clots, microvascular thrombosis and subsequent depletion of coagulation factors and platelets. [29]
32	**Vascular dysfunction**	Refers to the ability of the vascular system to ensure sufficient end-organ perfusion.	Coagulopathy, Electrolytes and metabolites imbalance, Acidosis	This is a concept node that summarizes all possible causes of impaired vascular function (all vascular related nodes).
33	**Ischemic cardiac injury**	Myocardial cell death due to insufficient cardiac perfusion to meet metabolic requirements.	Hyperdynamic circulation, Hypercoagulable state, Hypoxemia	The increased cardiac output observed in hyperdynamic circulation increases the metabolic requirements of myocardial cells. Thrombosis prevents blood from reaching the myocardial cells. Hypoxemia reduces the available oxygen in blood to meet metabolic requirements. [11], [30]

ID	Variable name	Description	Parent nodes	Relationships with parent nodes
34	**Acute cardiac inflammation**	Altered status of myocardial cells stimulated by chemical factors released by injured cells and immune cells.	Systemic immune/inflam. response, Direct viral injury	Direct viral injury damages myocardial cells. Cell damage and death and immune responses to that damage give rise to local inflammation [31], [32].
35	**Abnormal contractility**	Abnormal cardiac electrophysiology resulting in an alteration in the rate and/or coordination of contractility.	Ischemic cardiac injury, Acute cardiac inflammation	Inflammation and ischemia damage cells impeding normal electrical signaling and the coordination of cardiac contractility [32].
36	**Reduced stroke volume**	Reduced volume of blood ejected from the heart with each left ventricular contraction.	Reduced functional intravascular volume, Ischemic cardiac injury, Acute cardiac inflammation, Pulmonary hypertension, Hyperdynamic circulation, Abnormal contractility, Vascular resistance	The stroke volume is dependent on the volume of blood filling the left ventricle before contracting (systole). Reduced functional intravascular volume and pulmonary hypertension lower this volume. Hyperdynamic circulation reduces the time for filling to occur. Ischemic cardiac injury, acute cardiac inflammation and abnormal contractility reduce the volume ejected from the left ventricle with each contraction. Increased vascular resistance leads to reduced stroke volume via diminished ventricular compliance. [32]
37	**Reduced cardiac output**	A reduction in the volume of blood the heart pumps per unit time.	Abnormal contractility, Reduced stroke volume	Cardiac output is a function of heart rate and stroke volume. Reducing stroke volume decreases output. Abnormal contractility alters the heart rate [11].
38	**Cardiac failure**	Insufficient cardiac output to meet the body’s requirements.	Reduced cardiac output, Electrolytes and metabolites imbalance, Acidosis	Electrolytes and metabolites imbalance and acidosis impair the heart muscles contractility, reducing cardiac output. [33]
39	**Heart stops**	Complete arrest of cardiac contractility.	Respiration stops	Hypoxemia and poor perfusion cause cellular dysfunction. In the heart this leads to failure of electrical signaling and contractility.
40	**Liver dysfunction**	Impaired ability of liver to perform one or more of protein synthesis, metabolism of substrates including drugs, and bilious excretion of waste products.	Reduced supply of oxygen and metabolites, Systemic immune/ inflam. response, Direct viral injury	Viral damage, inflammation and poor nutrient supply damage and impair cellular function in the liver [34], [35].
41	**Liver failure**	Pathological inability of the liver to meet the needs of the body for protein synthesis, metabolism, and/or biliary excretion.	Liver dysfunction, Acidosis	Progression of liver dysfunction, which may both result from and contribute to acidosis, may lead to synthetic and metabolic function failure [36].
42	**Kidney dysfunction**	Impaired ability of kidney to control one or more of the body's fluid balance, appropriate electrolyte concentration or eliminate substrates.	Reduced supply of oxygen and metabolites, Systemic immune/ inflam. response, Direct viral injury	Direct damage, inflammation and lack of nutrients impair cellular function in the kidney, altering water and electrolyte balance capacity [37], [38].

ID	Variable name	Description	Parent nodes	Relationships with parent nodes
43	**Kidney failure**	Pathological inability of the kidney fails to meet the body's needs for fluid balance, maintenance of appropriate electrolyte concentrations and/or eliminate substrates.	Kidney dysfunction, Electrolytes and metabolites imbalance, Acidosis	Significant damage to the cells in the kidney resulting in loss of function. Acid-base, electrolyte and metabolite imbalance place competing strains on limited homeostasis capacity. [18]
44	**Hematologic dysfunction**	Impaired ability of bone marrow to produce blood cells.	Reduced supply of oxygen and metabolites, Systemic immune/ inflam. response, Direct viral injury	Viral damage, inflammation and poor nutrient supply damage and impair cellular function in the bone marrow [39].
45	**Hematologic failure**	Pathological inability of the bone marrow to produce sufficient blood cells to meet the body's needs.	Marrow dysfunction, Electrolytes and metabolites imbalance, Acidosis	Significant damage to the progenitor cells in the bone marrow through lack of nutrients and direct damage [39].
46	**Gut dysfunction**	Impaired ability of the gastrointestinal tract to digest food, absorb nutrients and/or excrete waste.	Reduced supply of oxygen and metabolites, Systemic immune response, Direct viral injury	Direct viral injury of enterocytes, systemic inflammation and hypoperfusion may impair gastrointestinal tract function [40].
47	**Gut failure**	Pathological inability of the gastrointestinal tract to meet the body's needs to digest food, absorb nutrients and/or excrete waste.	Gut dysfunction, Electrolytes and metabolites imbalance, Acidosis	Severe gastrointestinal dysfunction may result in reduction of enteric functions below the minimum necessary for absorption of macronutrients, water and electrolytes. The metabolic and electrolyte disturbances associated with systemic sepsis and inflammation may contribute to gut failure [41].
48	**Brainstem dysfunction**	Impaired ability of the brainstem to perform basic functions including brainstem reflexes, maintain the sleep-wake cycle and autonomic control of the cardiocirculatory and respiratory systems.	Systemic immune/inflam. response, Reduced supply of oxygen and metabolites	The brainstem is prone to vascular and inflammatory insults including compromised brainstem perfusion from impaired autoregulation of cerebral blood flow from sepsis; neuro-inflammatory processes from excessive systemic inflammatory response; and metabolic disturbance including electrolyte disturbance and renal or liver failure [42].
49	**Brainstem failure**	The complete loss of brainstem function.	Electrolytes and metabolites imbalance, Acidosis, Brainstem dysfunction	Complete cessation of brainstem functions with irreversible loss of consciousness and the capacity to breath, leading to cardiocirculatory and respiratory failure and death [42].
50	**Cortical brain dysfunction**	Impaired ability of the central nervous system to perform higher level functions including maintenance of consciousness and cognitive processes.	Systemic immune response, Reduced supply of oxygen and metabolites	Cerebral dysfunction due to the systemic inflammatory response to infection without direct central nervous system infection, mediated via excessive microglial activation, impaired cerebral perfusion, blood-brain-barrier dysfunction and altered neurotransmission. Severe hypoxemia and glucose dysregulation can potentiate dysfunction. [43]
51	**Coma**	A state of prolonged unresponsiveness with inability to respond appropriately to stimuli.	Liver dysfunction, Electrolytes and metabolites imbalance, Acidosis, Cortical brain dysfunction	Significant cerebral dysfunction from sepsis, metabolic and electrolyte disturbance may result in loss of higher level functions including consciousness[43]. Severe liver dysfunction may result in hyperammonemia with neuronal dysfunction and cerebral edema, with progression to coma [44].

Selected References

[1] W. Sungnak *et al.*, "SARS-CoV-2 entry factors are highly expressed in nasal epithelial cells together with innate immune genes," *Nature Medicine*, vol. 26, no. 5, Art. no. 5, May 2020, doi: 10.1038/s41591-020-0868-6.

[2] Z. Varga *et al.*, "Endothelial cell infection and endotheliitis in COVID-19," *The Lancet*, vol. 395, no. 10234, pp. 1417–1418, May 2020, doi: 10.1016/S0140-6736(20)30937-5.

[3] K. P. Y. Hui *et al.*, "Tropism, replication competence, and innate immune responses of the coronavirus SARS-CoV-2 in human respiratory tract and conjunctiva: an analysis in ex-vivo and in-vitro cultures," *The Lancet Respiratory Medicine*, vol. 8, no. 7, pp. 687–695, Jul. 2020, doi: 10.1016/S2213-2600(20)30193-4.

[4] F. Potus *et al.*, "Novel insights on the pulmonary vascular consequences of COVID-19," *Am J Physiol Lung Cell Mol Physiol*, vol. 319, no. 2, pp. L277–L288, Aug. 2020, doi: 10.1152/ajplung.00195.2020.

[5] R. T. Dhawan *et al.*, "Beyond the clot: perfusion imaging of the pulmonary vasculature after COVID-19," *The Lancet Respiratory Medicine*, vol. 9, no. 1, pp. 107–116, Jan. 2021, doi: 10.1016/S2213-2600(20)30407-0.

[6] Y.-C. Lai, K. C. Potoka, H. C. Champion, A. L. Mora, and M. T. Gladwin, "Pulmonary Arterial Hypertension: The Clinical Syndrome," *Circ Res*, vol. 115, no. 1, pp. 115–130, Jun. 2014, doi: 10.1161/CIRCRESAHA.115.301146.

[7] C. Brusasco *et al.*, "Continuous positive airway pressure in COVID-19 patients with moderate-to-severe respiratory failure," *European Respiratory Journal*, vol. 57, no. 2, Feb. 2021, doi: 10.1183/13993003.02524-2020.

[8] H. Chu *et al.*, "Comparative Replication and Immune Activation Profiles of SARS-CoV-2 and SARS-CoV in Human Lungs: An Ex Vivo Study With Implications for the Pathogenesis of COVID-19," *Clinical Infectious Diseases*, vol. 71, no. 6, pp. 1400–1409, Sep. 2020, doi: 10.1093/cid/ciaa410.

[9] S. Baron, M. Fons, and T. Albrecht, "Viral Pathogenesis," in *Medical Microbiology*, 4th ed., S. Baron, Ed. Galveston (TX): University of Texas Medical Branch at Galveston, 1996. Accessed: Sep. 20, 2021. [Online]. Available: http://www.ncbi.nlm.nih.gov/books/NBK8149/

[10] J. Gu and C. Korteweg, "Pathology and Pathogenesis of Severe Acute Respiratory Syndrome," *The American Journal of Pathology*, vol. 170, no. 4, pp. 1136–1147, Apr. 2007, doi: 10.2353/ajpath.2007.061088.

[11] P. E. Bickler, J. R. Feiner, M. S. Lipnick, and W. McKleroy, "'Silent' Presentation of Hypoxemia and Cardiorespiratory Compensation in COVID-19," *Anesthesiology*, Sep. 2020, doi: 10.1097/ALN.0000000000003578.

[12] S. Dhont, E. Derom, E. Van Braeckel, P. Depuydt, and B. N. Lambrecht, "The pathophysiology of 'happy' hypoxemia in COVID-19," *Respiratory Research*, vol. 21, no. 1, p. 198, Jul. 2020, doi: 10.1186/s12931-020-01462-5.

[13] S. Caravita *et al.*, "Haemodynamic characteristics of COVID-19 patients with acute respiratory distress syndrome requiring mechanical ventilation. An invasive assessment using right heart catheterization," *European Journal of Heart Failure*, vol. 22, no. 12, pp. 2228–2237, 2020, doi: 10.1002/ejhf.2058.

[14] C. Chelazzi, G. Villa, P. Mancinelli, A. R. De Gaudio, and C. Adembri, "Glycocalyx and sepsis-induced alterations in vascular permeability," *Critical Care*, vol. 19, no. 1, p. 26, Dec. 2015, doi: 10.1186/s13054-015-0741-z.

[15] "Full article: A multidisciplinary consensus on dehydration: definitions, diagnostic methods and clinical implications." https://www.tandfonline.com/doi/full/10.1080/07853890.2019.1628352 (accessed Sep. 20, 2021).

[16] "Physiology, Peripheral Vascular Resistance - StatPearls - NCBI Bookshelf." https://www.ncbi.nlm.nih.gov/books/NBK538308/ (accessed Sep. 24, 2021).

[17] G. Lippi, A. M. South, and B. M. Henry, "Electrolyte imbalances in patients with severe coronavirus disease 2019 (COVID-19)," *Ann Clin Biochem*, vol. 57, no. 3, pp. 262–265, May 2020, doi: 10.1177/0004563220922255.

[18] P. Rossignol, A. J. Coats, O. Chioncel, I. Spoletini, and G. Rosano, "Renal function, electrolytes, and congestion monitoring in heart failure," *European Heart Journal Supplements*, vol. 21, no. Supplement_M, pp. M25–M31, Dec. 2019, doi: 10.1093/eurheartj/suz220.

[19] A. M. Deane, M. J. Chapman, A. R. Blaser, S. A. McClave, and A. Emmanuel, "Pathophysiology and Treatment of Gastrointestinal Motility Disorders in the Acutely Ill," *Nutrition in Clinical Practice*, vol. 34, no. 1, pp. 23–36, 2019, doi: 10.1002/ncp.10199.

[20] J. A. Kraut and N. E. Madias, "Metabolic acidosis: pathophysiology, diagnosis and management," *Nature Reviews Nephrology*, vol. 6, no. 5, Art. no. 5, May 2010, doi: 10.1038/nrneph.2010.33.

[21] A. Kimmoun, E. Novy, T. Auchet, N. Ducrocq, and B. Levy, "Hemodynamic consequences of severe lactic acidosis in shock states: from bench to bedside," *Crit Care*, vol. 19, p. 175, Apr. 2015, doi: 10.1186/s13054-015-0896-7.

[22] J.-H. Lim *et al.*, "Hypertension and Electrolyte Disorders in Patients with COVID-19," *Electrolyte Blood Press*, vol. 18, no. 2, pp. 23–30, Dec. 2020, doi: 10.5049/EBP.2020.18.2.23.

[23] M. Dolhnikoff *et al.*, "Pathological evidence of pulmonary thrombotic phenomena in severe COVID-19," *Journal of Thrombosis and Haemostasis*, vol. n/a, no. n/a, doi: 10.1111/jth.14844.

[24] Y. Sakr *et al.*, "The clinical spectrum of pulmonary thromboembolism in patients with coronavirus disease-2019 (COVID-19) pneumonia: A European case series," *J Crit Care*, vol. 61, pp. 39–44, Feb. 2021, doi: 10.1016/j.jcrc.2020.09.021.

[25] L. C. Price, C. McCabe, B. Garfield, and S. J. Wort, "Thrombosis and COVID-19 pneumonia: the clot thickens!," *European Respiratory Journal*, vol. 56, no. 1, Jul. 2020, doi: 10.1183/13993003.01608-2020.

[26] A. de Matthaeis *et al.*, "Effects of hypercapnia on peripheral vascular reactivity in elderly patients with acute exacerbation of chronic obstructive pulmonary disease," *Clin Interv Aging*, vol. 9, pp. 871–878, May 2014, doi: 10.2147/CIA.S57548.

[27] "Cerebral Circulation: XII. The Effect on Pial Vessels of Variations in The Oxygen and Carbon Dioxide Content of The Blood | Archives of Neurology & Psychiatry | JAMA Network." https://jamanetwork.com/journals/archneurpsyc/article-abstract/644583 (accessed Sep. 24, 2021).

[28] C. K. Chan and P. M. Vanhoutte, "Hypoxia, vascular smooth muscles and endothelium," *Acta Pharmaceutica Sinica B*, vol. 3, no. 1, pp. 1–7, Feb. 2013, doi: 10.1016/j.apsb.2012.12.007.

[29] M. Giustozzi *et al.*, "Coagulopathy and sepsis: Pathophysiology, clinical manifestations and treatment," *Blood Reviews*, p. 100864, Jun. 2021, doi: 10.1016/j.blre.2021.100864.

[30] A. R. Chapman, P. D. Adamson, and N. L. Mills, "Assessment and classification of patients with myocardial injury and infarction in clinical practice," *Heart*, vol. 103, no. 1, pp. 10–18, Jan. 2017, doi: 10.1136/heartjnl-2016-309530.

[31] Tucker Nathan R. *et al.*, "Myocyte-Specific Upregulation of ACE2 in Cardiovascular Disease," *Circulation*, vol. 142, no. 7, pp. 708–710, Aug. 2020, doi: 10.1161/CIRCULATIONAHA.120.047911.

[32] "Covid-19 and the cardiovascular system: a comprehensive review | Journal of Human Hypertension." https://www.nature.com/articles/s41371-020-0387-4 (accessed Sep. 20, 2021).

[33] F. Bader, Y. Manla, B. Atallah, and R. C. Starling, "Heart failure and COVID-19," *Heart Fail Rev*, vol. 26, no. 1, pp. 1–10, Jan. 2021, doi: 10.1007/s10741-020-10008-2.

[34] J. Wu, S. Song, H.-C. Cao, and L.-J. Li, "Liver diseases in COVID-19: Etiology, treatment and prognosis," *World J Gastroenterol*, vol. 26, no. 19, pp. 2286–2293, May 2020, doi: 10.3748/wjg.v26.i19.2286.

[35] "COVID-19 and Liver Dysfunction: Current Insights and Emergent Therapeutic Strategies." https://www.ncbi.nlm.nih.gov/pmc/articles/PMC7132016/ (accessed Sep. 24, 2021).

[36] A. Drolz *et al.*, "Acid–base status and its clinical implications in critically ill patients with cirrhosis, acute-on-chronic liver failure and without liver disease," *Annals of Intensive Care*, vol. 8, no. 1, p. 48, Apr. 2018, doi: 10.1186/s13613-018-0391-9.

[37] J. Watchorn, D. Y. Huang, J. Joslin, K. Bramham, and S. D. Hutchings, "Critically Ill COVID-19 Patients with Acute Kidney Injury Have Reduced Renal Blood Flow and Perfusion Despite Preserved Cardiac Function: A Case-Control Study Using Contrast-Enhanced Ultrasound," *Shock*, vol. 55, no. 4, pp. 479–487, Apr. 2021, doi: 10.1097/SHK.0000000000001659.

[38] F. Braun *et al.*, "SARS-CoV-2 renal tropism associates with acute kidney injury," *The Lancet*, vol. 0, no. 0, Aug. 2020, doi: 10.1016/S0140-6736(20)31759-1.

[39] "Bone Marrow and Peripheral Blood Findings in Patients Infected by SARS-CoV-2 | American Journal of Clinical Pathology | Oxford Academic." https://academic.oup.com/ajcp/article/155/5/627/6121360 (accessed Sep. 20, 2021).

[40] A. Reintam Blaser, J. Gunst, and Y. M. Arabi, "The gut in COVID-19," *Intensive Care Med*, vol. 47, no. 9, pp. 1024–1027, Sep. 2021, doi: 10.1007/s00134-021-06461-8.

[41] L. Pironi *et al.*, "ESPEN endorsed recommendations. Definition and classification of intestinal failure in adults," *Clinical Nutrition*, vol. 34, no. 2, pp. 171–180, Apr. 2015, doi: 10.1016/j.clnu.2014.08.017.

[42] S. Benghanem *et al.*, "Brainstem dysfunction in critically ill patients," *Critical Care*, vol. 24, no. 1, p. 5, Jan. 2020, doi: 10.1186/s13054-019-2718-9.

[43] R. Sonneville *et al.*, "Understanding brain dysfunction in sepsis," *Ann. Intensive Care*, vol. 3, no. 1, p. 15, May 2013, doi: 10.1186/2110-5820-3-15.

[44] "Hepatic Encephalopathy | NEJM." https://www.nejm.org/doi/full/10.1056/NEJMra1600561 (accessed Sep. 24, 2021).
