# Supplementary material for: Modeling COVID-19 disease processes by remote elicitation of causal Bayesian networks from medical experts
Source: BMC Med Res Methodol. 2023 Mar 29;23:76. doi: 10.1186/s12874-023-01856-1 (PMC10050813; doi:10.1186/s12874-023-01856-1)
Supplement: Supplementary file 5 — Additional file 5. Members of COVID BN Advisory Group v1.2. This table lists all the members of our COVID BN Advisory Group who opted to be individually acknowledged, with their institutions and relevant qualifications. [file 12874_2023_1856_MOESM5_ESM.pdf]

Members of COVID BN Advisory Group v1.2

The following members agreed to be acknowledged individually; others preferred to remain anonymous. We thank all members for participating in the elicitation sessions, including group workshops, one-on-one meetings, and surveys. This supplement was prepared for Mascaro et al (2022); reuse freely with acknowledgement.

Name	Institutions	Qualifications
Alex Semprini	Medical Research Institute of New Zealand, NZ	MBBS, BSc (Hons), MInstD, PhD
Andrew Martin	Department of General Paediatrics, Perth Children's Hospital, AU<br>University of Western Australia, AU	MBBS, FRACP, MD
Andrew McLean-Tooke	Department of Clinical Immunology, Sir Charles Gairdner Hospital, AU<br>Department of Laboratory Immunology, PathWest QEII Medical Centre, AU	BSc, MBChB, MD, FRCP, FRCPath FRCPA, FRACP
Ben Marais	University of Sydney Institute for Infectious Diseases, AU	MD, PhD
Benjamin Tang	Nepean Hospital, AU<br>Westmead Institute for Medical Research, AU	PhD, FCICM, MBBS, MMed (Clin Epi)
Charlie McLeod	Infectious Diseases Department, Perth Children's Hospital, AU<br>Infectious Diseases Implementation Research Team, Telethon Kids Institute, AU	MBBS (Hons), FRACP, DTM&H, PhD
Christopher Blyth	Faculty of Health and Medical Sciences, The University of Western Australia, AU<br>Department of Infectious Diseases, Perth Children's Hospital, AU<br>Department of Microbiology, PathWest Laboratory Medicine WA, QEII Medical Centre, AU<br>Wesfarmers Centre for Vaccines and Infectious Diseases, Telethon Kids Institute, AU<br>National Health and Medical Research Council, AU	MBBS (Hons), DCH, FRACP FRCPA, PhD
Chris Lemoh	Department of Medicine, School of Clinical Sciences at Monash Health, Monash University, AU	MBBS, PhD, FRACP
Claire Waddington	Department of Medicine, University of Cambridge, UK	MBBS, DPhil
David Nolan	Department of Immunology, Royal Perth Hospital, AU	MBBS, FRACP, PhD
Edward Raby	Department of Infectious Diseases, Fiona Stanley Hospital, AU<br>Department of Microbiology, PathWest, Royal Perth Hospital, AU	MBBS, FRACP, FRCPA
Gladymar Perez	Curtin University, AU<br>Telethon Kids Institute, AU	MD, DCTM&H
Guy Marks	University of New South Wales Sydney, AU<br>Woolcock Institute of Medical Research, AU	BMedSc, MB, BS, PhD, MRCP, FRACP, FAFPHM, FAHMS

Name	Institutions	Qualifications
Justin Denholm	Victorian Infectious Diseases Service, Royal Melbourne Hospital, Melbourne Health, AU<br>Department of Infectious Diseases, University of Melbourne at the Peter Doherty Institute for Infection and Immunity, AU	BMed, MBioethics, MPH+TM, PhD, FRACP
Lisa Pilgram	Lean European Open Survey on SARS-CoV‐2 infected patients (LEOSS), DE	Physician
Nicholas Anstey	Menzies School of Health Research, AU<br>Royal Darwin Hospital, AU	MBBS (Hons), PhD, FRACP
Magdalena Plebanski	School of Health and Biomedical Sciences, RMIT University, AU	BScHon, DPS, MBA, PhD
Mark Boyd	Faculty of Health and Medical Sciences, University of Adelaide, AU	MD, FRACP
Meredith Borland	Perth Children's Hospital Emergency Department, AU<br>Divisions of Emergency Medicine and Paediatrics, School of Medicine, University of Western Australia, AU	MBBS, FRACGP, FACEM
Michael Maze	University of Otago, NZ	MB, ChB, DCH, DTM&H, PhD, FRACP
Mina John	Department of Immunology, Clinipath Pathology, AU<br>Department of Clinical Immunology, Royal Perth Hospital, AU	MBBS, FRACP, FRCPA
Paul Middleton	South Western Emergency Research Institute, Liverpool Hospital, AU<br>South Western Sydney Clinical School, University of New South Wales, AU	RGN, MBBS, DipIMCRCS (Ed), MMed (Clin Epi),<br>MD (Imp Lond), FRCS (Eng), FACPara, FRCEM, FACEM
Simon Craig	Department of Paediatrics, Monash University, AU<br>Paediatric Emergency Department, Monash Medical Centre, Emergency Service, Monash Health, AU	MBBC, FACEM, MPH, MHPE
Steve Y.C. Tong	Department of Infectious Diseases, The University of Melbourne at the Peter Doherty Institute for Infection and Immunity, AU<br>Victorian Infectious Diseases Service, The Royal Melbourne Hospital, at the Peter Doherty Institute for Infection and Immunity, AU	MBBS, PhD
Susan Benson	School of Pathology and Laboratory Medicine, University of Western Australia, AU<br>School of Population Health, Curtin University, AU	MBBS, FRACP, FRCPA, FACHSM, DTM&H DipClinEpi
Toby Richards	Covid Research Response Trial, University of Western Australia, AU	MD, FRCS
